# Supplementary material for: Gender and Socioeconomic Differences in the Prevalence and Patterns of Multimorbidity among Middle-Aged and Older Adults in China
Source: Int J Environ Res Public Health. 2022 Dec 16;19(24):16956. doi: 10.3390/ijerph192416956 (PMC9779237; doi:10.3390/ijerph192416956)
Supplement: Supplementary file 1 [file ijerph-19-16956-s001.zip › ijerph-2056676-supplementary.pdf]

Table S1. Model-fit statistics comparison for latent class analysis

	AIC ↓	BIC ↓	aBIC	VLMR-LRT	BLRT	Entropy ↑	proportion
CLASS1	204280.272	204390.609	204346.118	—	—	—	—
CLASS2	194726.761	194955.315	194863.155	0.0000	0.0000	0.629	0.30651/ 0.69349
CLASS3	192705.259	193052.031	192912.201	0.0000	0.0000	0.682	0.19311/0.13022/0.67667
**CLASS4**	**191767.533**	**192232.523**	**192045.024**	**0.0039**	**0.0040**	**0.718**	**0.65024/ 0.06386/ 0.09995/ 0.18595**
CLASS5	190896.725	191479.933	191244.764	0.0000	0.0000	0.657	0.03942/ 0.03794/ 0.55136/ 0.16836/ 0.20292
CLASS6	190691.701	191393.126	191110.289	0.0167	0.0172	0.639	0.17051/ 0.16136/ 0.03625/ 0.04775/ 0.03932/ 0.54481

AIC Akaike Information Criterion; BIC Bayesian Information Criterion; aBIC Adjusted Bayesian Information Criterion; LMR Lo-Mendell-Rubin Likelihood Ratio Test; BLRT Bootstrap Likelihood Ratio Test

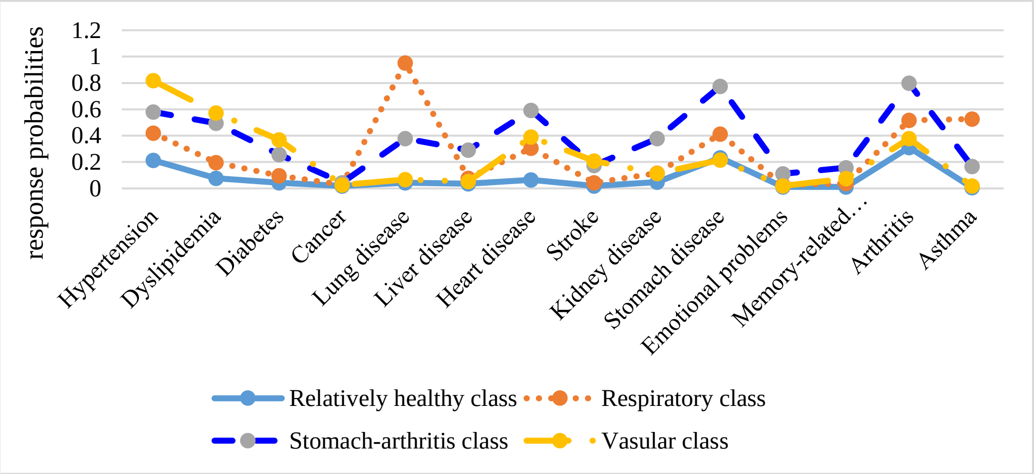

Figure S1. Four class model of multimorbidity patterns.
